# Fincher syndrome: insights into a rare neurosurgical condition

**DOI:** 10.1007/s10143-024-02828-9

**Published:** 2024-09-10

**Authors:** Muhammad Kashif, Alan Hernández-Hernández, Oday Atallah

**Affiliations:** 1 Faculty of medicine, Midwestern University, Glendale, AZ USA; 2 Department of Neurosurgery, National Institute of Neurology and Neurosurgery, Mexico City, Mexico; 3 Departemnt of Neurosurgery, Hannover Medical School, Hannover, Germany

**Keywords:** Fincher syndrome, Spinal cord tumor, Subarachnoid hemorrhage

## Introduction

Tuleasca et al. (2020) describes Fincher syndrome as an extremely rare and relatively understudied neurological condition that is defined by the simultaneous development of spinal subarachnoid hemorrhage (SAH) and spinal cord tumors [14, 16]. It was named after a scholar known as Dr. Fincher who was the first to offer details of the connection between the two conditions in mid-twentieth century [3]. According to Moore et al. (2018), the condition is an indication of how vascular complications and neoplastic processes intersect within the central nervous system [11]. Although the two conditions hardly occur together, Tuleasca et al. (2020) argues that it has been observed in 5 to 10 per cent of SAH patients, resulting in severe complications [16]. Since early detection and prompt intervention can drastically influence the prognosis of this uncommon syndrome, it is imperative to understand the diagnosis, pathophysiology, clinical presentation, and potential treatment choices to achieve better patient outcomes.

## Pathophysiology

**Fig. 1:**
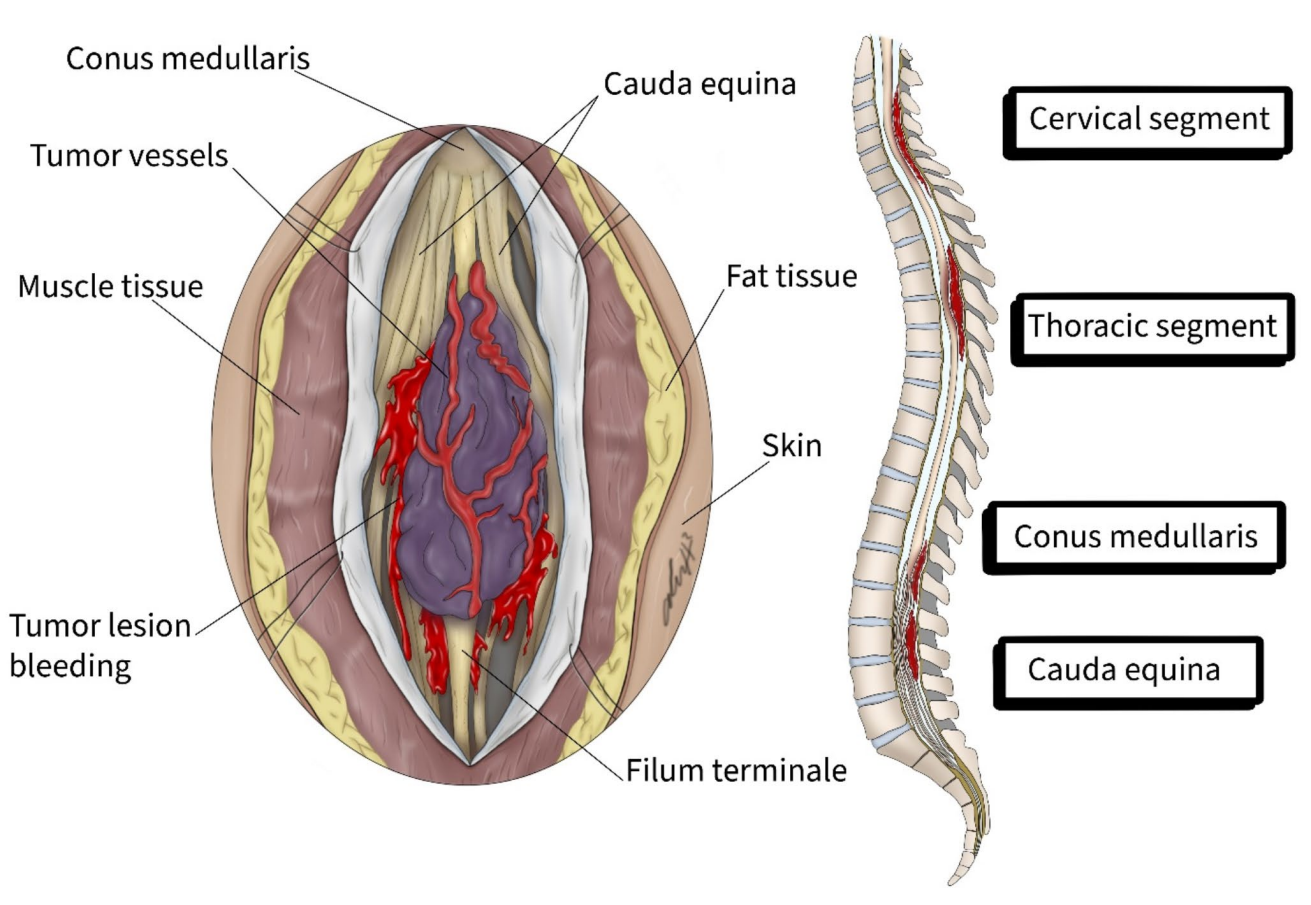
An illustration demonstrates the neurosurgical condition of Fincher syndrome

There exists a huge knowledge gap of the syndrome’s pathophysiology. However, several concepts have been brought up to unravel the syndrome’s causative factors and the link between the two conditions. Fincher et al. (2015) suggests vascular disruption as the first approach to explain the syndrome’s pathophysiology [3]. He contends that tumors growing in the spinal cord usually surrounded by numerous blood vessels and as they continue to enlarge, they obstruct normal blood flow and results in rupture and subsequently bleeding [11]. The disruption may also arise from the tumor having excessive blood supply causing it to rapture. When the tumor grows larger than the space around it, or the blood vessels succumb to tumor pressure, it results in ischemia and then hemorrhage (Fig. 1).

Anderson and Patel (2019) also argue that the syndrome may be explained by the tumor’s mass effect on surrounding structures within the spinal cord causing mechanical compression which damages delicate capillaries [1]. This can take place in cases where a tumor grows larger and larger yet it is confined in a small space such as thoracic and cervical vertebral canals.

According to some researchers, subarachnoid hemorrhage likely occurs when the venous system in the spinal cord is exposed to excessive pressure [10, 13, 16]. The elevated pressure on the vessels causes them to burst resulting in hemorrhage. In some cases, the syndrome has been attributed to vascular defects within the spinal cord [3]. For instance, patient with arteriovenous malformations is prone to developing tumors and experiencing hemorrhage. The risk of hemorrhage is particularly high among spinal cord tumor patients who have arteriovenous malformations.

## Clinical presentation

The syndrome’s clinical indicators vary from one patient to another, primarily because the tumors differ in dimensions and location as well as the degree of bleeding observed. Patient’s usually present with sudden, acute sciatica, lower back pain and headache. Some neurological malfunctions such as numbness, weakness of muscles, and in extreme cases paralysis may be observed and may vary based on the size and location of the tumor [5].

According to Brown and Roberts (2019), severe occurrences of this condition may cause patients to have problems with their bladder function which emerges from the compression of the spinal cord [2]. Besides, Garcia and Wilson (2020) assert that meningeal irritation may be manifested through stiffness in the neck, sensitivity to light, changes in reflexes, and feelings of nausea [4]. These symptoms may overlap, and hence, it is prudent to commence additional investigation, particularly if the patient has no history of trauma or recognized vascular complications.

## Diagnosis

The diagnosis of Fincher Syndrome, especially for patients with a recognized spinal cord tumor, who experience abrupt and intense symptoms of neurological disease, requires tools such as magnetic resonance imaging (MRI), computed tomography (CT), lumbar puncture, angiography and biopsy [12]. The MRI is the most dependable technique for visualizing spinal cord tumors and the bleeding that may ensure. Spinal cord tumors such as metastases, astrocytoma, and ependymomas can be distinguished from one another through the use of gadolinium enhanced MRI [7, 17]. Kuo (2019) suggests that physicians may use T1- and T2-weighted scans to determine whether there is hemorrhage within the tumor or the surrounding space and to what extent it has occurred [8]. They may also opt for CT myelography to determine if there is any tumors or hemorrhage. Kwan (2014) asserts that when looking out for sign of hemorrhage it is prudent to consider lumbar puncture which may be signified by xanthochromia or excessive red blood cell count [9]. However, Moore et al. (2018) caution that lumbar puncture tests should be carried out with great caution since it has the potential to worsen neurological defects where a spinal cord tumor is present [11].

In stances where a physician confirms vascular defects, Tuleasca et al. (2020) suggests they should advise patient to undertake spinal angiography to determine if defects such as retinovascular malformations could be present and causing the bleeding [16]. This test is essential in determining and confirming the existence of vascular defects and hence informs the choice of treatment plan. Taylor (2018) also suggests that physicians should also carry out biopsy evaluations to ascertain the histological nature of the tumor before they commence treatment [15]. A biopsy test, which is typically carried out using minimally invasive techniques guided by CT and MRI scans, supports the diagnosis and informs the choice of a treatment plan.

Because a few other conditions have similar anatomical symptoms with Fincher syndrome, it is prudent for physicians to ascertain the diagnosis through the aforementioned test before they commence treatment.

## Treatment

The treatment of Fincher Syndrome begins by managing both the tumor and hemorrhage, aimed at stabilizing the patient and avert any neurological deterioration. Such stabilization options may include immobilization, analgesics, and in some instances, corticosteroids to decrease inflammation and swelling around the spinal cord [14]. Some of the treatment strategies include surgical resection, radio therapy, chemotherapy, hemorrhage management, and rehabilitation. Surgical resection is usually the primary treatment option for accessible spinal cord tumors which intends to excise to the greatest extent feasible when minimizing harm to the adjacent spinal cord [2]. Further, decompressive surgery is necessary if the tumor severely compressing the spinal cord in order to release pressure and stop the condition from getting worse. The selection of surgical methods, such as laminectomy and endoscopic resection, depends on the tumor’s size, specific location, histological classification, and the patient’s overall state of health [6]. Alternative treatment choices include radiation therapy which is considered if the patient is not deemed suitable for surgery or if the tumor is not operable. Another alternative approach is stereotactic radiosurgery that uses high radiation doses to target the tumor while exposing nearby tissues to as little radiation as possible [4]. Besides, chemotherapy is applied to definite types of tumors where systemic treatment is indicated.

Notably, proper care after surgery is crucial for achieving the best outcomes and reducing the risk of complications. Jones et al. (2021) argues that patients require proper rehabilitation care after treatment to restore their neurological functions [7]. Physicians also need to continuously monitor patient’s progress to promptly detect any tumor growth or recurrence [16]. The treatment choices may differ depending on the dimensions of the tumor, the time of intervention, and the extent of neurological deterioration when the patient is diagnosed.

## Prognosis

Patients tend to record diverse prognoses depending on the size and location of the tumor, the level of hemorrhage and time of intervention. Tuleasca et al. (2021) observes that the overall rate of survival is about 80 per cent at five years after treatment [16]. This statistic underscores the relevance of timely diagnosis and prompt intervention in enhancing outcomes. The outcomes are projected to be better in cases where the tumor is benign and can be excised through surgical intervention, allowing quicker recovery and restoration of neurological capabilities. Nevertheless, when the tumor is malignant, or hemorrhage is extensive, the prognosis becomes more uncertain, as there is a greater likelihood of enduring neurological impairments or even fatality [10]. Further, patients face a great risk of tumor or hemorrhage recurrence when surgical interventions do not achieve total excision. It is therefore important for healthcare providers to follow up, recommend imaging reviews so as to notice any recurrence early enough to allow appropriate interventions. When tumor residuals are detected or tumors recur, physicians may opt for radiation therapy or chemotherapy.

## Conclusion

Fincher syndrome is a very rare and relatively understudied neurological condition that is defined by the simultaneous development of spinal subarachnoid hemorrhage and spinal cord tumors. Although it is considered relatively rare, it poses fatal repercussions when diagnosed late or appropriate interventions are not administered. Early detection and timely interventions are crucial factors in determining patient outcomes for this condition. Physicians should suspect this condition in patient who complain of sudden, acute pain, that may spread from the lower back to the limbs, deteriorating neurological defects, and symptoms of subarachnoid hemorrhage and more importantly if the patient does not have a history of trauma. Although research of this syndrome has made some progress, it is principally insufficient. Hence, there is great need to conduct further studies regarding diagnostic, imaging, surgical, and therapeutic mechanisms of this syndrome and improve patient outcomes and survival rates.

## Data Availability

No datasets were generated or analysed during the current study.
